# The mitochondrial genome of *Globodera ellingtonae* is composed of two circles with segregated gene content and differential copy numbers

**DOI:** 10.1186/s12864-016-3047-x

**Published:** 2016-09-05

**Authors:** Wendy S. Phillips, Amanda M. V. Brown, Dana K. Howe, Amy B. Peetz, Vivian C. Blok, Dee R. Denver, Inga A. Zasada

**Affiliations:** 1 Horticultural Crops Research Laboratory, Agricultural Research Service, United States Department of Agriculture, Corvallis, OR USA; 2 Department of Integrative Biology, Oregon State University, Corvallis, OR USA; 3 Cell and Molecular Sciences Group, Dundee Effector Consortium, James Hutton Institute, Dundee, UK

**Keywords:** Mitochondrial genomics, Multipartite mitochondrial genome, *Globodera*, Nematode, Replication, Transcription

## Abstract

**Background:**

The evolution of animal mitochondrial (mt) genomes has resulted in a highly conserved structure: a single compact circular chromosome approximately 14 to 20 kb long. Within the last two decades exceptions to this conserved structure, such as the division of the genome into multiple chromosomes, have been reported in a diverse set of metazoans. We report on the two circle multipartite mt genome of a newly described cyst nematode, *Globodera ellingtonae*.

**Results:**

The *G. ellingtonae* mt genome was found to be comprised of two circles, each larger than any other multipartite circular mt chromosome yet reported, and both were larger than the single mt circle of the model nematode *Caenorhabditis elegans*. The genetic content of the genome was disproportionately divided between the two circles, although they shared a ~6.5 kb non-coding region. The 17.8 kb circle (mtDNA-I) contained ten protein-coding genes and two tRNA genes, whereas the 14.4 kb circle (mtDNA-II) contained two protein-coding genes, 20 tRNA genes and both rRNA genes. Perhaps correlated with this division of genetic content, the copy number of mtDNA-II was more than four-fold that of mtDNA-I in individual nematodes. The difference in copy number increased between second-stage and fourth-stage juveniles.

**Conclusions:**

The segregation of gene types to different mt circles in *G. ellingtonae* could provide benefit by localizing gene functional types to independent transcriptional units. This is the first report of both two-circle and several-circle mt genomes within a single genus. The differential copy number associated with this multipartite mt organization could provide a model system for deconstructing mechanisms regulating mtDNA copy number both in somatic cells and during germline development.

**Electronic supplementary material:**

The online version of this article (doi:10.1186/s12864-016-3047-x) contains supplementary material, which is available to authorized users.

## Background

The structure of metazoan mitochondrial (mt) genomes is generally highly conserved as a single compact circle, most commonly between 14 and 20 kb [1]. Almost all metazoan mt genomes are composed of the same 12 to 13 electron transport chain (ETC) protein-coding genes, and the two ribosomal RNA (rRNA) and 22 transfer RNA (tRNA) genes used for the synthesis of those proteins. The order of these genes, however, is highly variable and sometimes even radically different between species in the same genus [1]. Nonetheless, individual genes must remain functionally intact for effective energy production by mitochondria. Both small (e.g. point mutations) and large abnormalities (e.g. large-scale deletions) in mt genomes are known contributors to various human diseases, such as maternally-inherited Leigh syndrome and Kearns-Sayre syndrome [2]. Despite the small size of metazoan mt genomes relative to nuclear genomes, much remains unknown regarding the processes of mt genome replication, transcription, and maintenance. There are two leading theories to explain the mode of mtDNA replication in animals: an asynchronous strand displacement model and a strand-coupled bidirectional replication model [3, 4]. More recently, other methods of replication have been proposed, including bootlace and rolling circle models [5, 6]. By investigating cases of unusual metazoan mtDNA structure, we may gain insight into the maintenance of normal mtDNA and factors leading to disease associated abnormalities.

Despite the highly conserved single circle mt genome structure throughout metazoa, structural variants have been discovered in a diverse set of species. The genomes of an isopod (*Armadillidium vulgare*) and several genera of Cnidarians have become linearized, sometimes then forming dimeric circles [7, 8]. For some organisms, with either linear or circular mtDNA, the genome has become multipartite, divided into two or more chromosomes. Shao et al. [9] introduced the terminology “mitochondrial karyotype” to describe the gene arrangement, topology (linear or circular), and number of chromosomes in multipartite mt genomes. A mt karyotype of a genome with two distinct circles has been reported in a small set of metazoans: thrips, booklice, rotifers, and nematodes. Within the thrip *Scirtothrips dorsalis* cryptic species complex, a South Asian species has two very different sized circles, 14.3 and 0.9 kb, whereas an East Asian species has just a single complete circle [10]. Booklice in the genus *Liposcelis*, rotifers in the genus *Brachionus*, and a nematode in the genus *Rhabditophanes* have mt genomes with two circles of similar size, ~8 to 13 kb [11–13]. The most highly studied group of organisms with multipartite circles are the mammalian blood-sucking lice in the suborder Anoplura, which have from nine to 20 circles of relatively similar sizes ranging from ~2 to 5 kb [14–18]. Recently, a similar multipartite arrangement was found in the closely related chewing lice suborder Rhynchophthirina [9]. The first metazoan circular multipartite genome to be reported was in a potato cyst nematode (PCN), *Globodera pallida*, which was shown to have at least six mtDNA circles ranging in size from ~6 to 9 kb [19]. Later the other known PCN, *G. rostochiensis*, was also reported to have multiple circles of a similar size range [20].

Recently a new species of *Globodera* (class: Chromadorea; order: Tylenchida) was described, *G. ellingtonae*, that is phylogenetically intermediate between these two PCN species [21, 22]. Given that PCN causes damage to potato crops throughout the world [23], the biology of this new PCN-like species is of considerable economic interest. It has a life cycle of approximately 70 days, with developmental rate dependent on temperature, and reproduces very successfully on potato [24, 25]. Investigations into its pathogenicity to potato (*Solanum tuberosum*) are ongoing (W Phillips and I Zasada, unpublished). Although multipartite mt genomes are not unique to parasites, and most reported parasite mt genomes are not multipartite, a large proportion of the reported multipartite circular genomes in bilateria are in parasites [9–20]. Given this, a focal question is how multipartite mt genomes might interplay with the biology and evolution of pathogenicity in *Globodera*. To begin to address that question, and as part of a broader genomic analysis of *G. ellingtonae*, we investigated the structure of the mt genome of this nematode.

We found *G. ellingtonae* has a multipartite mt genome of two extraordinarily large circles that occur at different copy numbers and with segregated gene content. We compare the mt genome structure of *G. ellingtonae* with that of other metazoans with multipartite circular genomes. However, we exclude from analysis comparisons with the gene order and non-coding sequence of the mt genomes of *G. pallida* and *G. rostochiensis*. Those comparisons, along with substantial corrections to published sequences, will be made in a subsequent publication (W Phillips, S Eves-van den Akker, and V Blok, unpublished). Nonetheless, data presented here uncovers a unique feature in the evolution of multipartite mt genomes in the genus *Globodera*: this single genus has species with mt genomes consisting of both two and several mt circles.

## Results

### Initial assembly, PCR, and Southern blot indicate two mtDNA circles

**Fig. 1:**
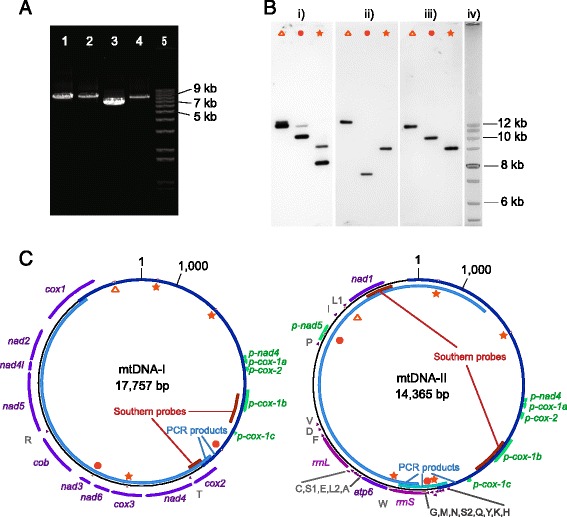
Mitochondrial (mt) genome organization in *Globodera ellingtonae*. **a** PCR amplification products of mtDNA used in cloning and sequencing: amplicons of 1) ‘outward’ amplicon of mtDNA-I, 2) ‘inward’ amplicon of mtDNA-I, 3) ‘outward’ amplicon of mtDNA-II, 4) ‘inward’ amplicon of mtDNA-II, and 5) 1 kb plus DNA ladder (Invitrogen). **b** Southern blots confirm the size and organization of the two circles. Symbols above each lane correspond to those in panel **c** and represent the restriction enzymes used to digest total DNA: a star for EcoRI, a triangle for KpnI, and a circle for PacI. Blots hybridized with (*i*) probe to shared sequence, exposure 1 min; (*ii*) probe to mtDNA-I specific sequence, exposure 4 min; (*iii*) probe to mtDNA-II specific sequence, exposure 30 s; and (*iv*) image of 1 kb plus ladder from ethidium bromide stained gel aligned to x-ray images. **c** Organization of *G. ellingtonae* mitochondrial genome. Protein-coding, rRNA, and pseudo- gene abbreviations are located next to arced arrows showing their location and direction. Single letter abbreviations of amino acids denote locations of associated tRNA genes. Locations of PCR products used in cloning and sequencing the circles and of probes used in Southern hybridizations are shown. Locations of nucleotides 1 and 1000 are noted on each circle for size reference. The *star*, *triangle*, and *square* indicate restriction enzyme sites as denoted above

We began by examining an initial assembly of genomic (nuclear and mitochondrial) Illumina MiSeq reads for mtDNA. Using individual *Heterodera glycines* and *Globodera pallida* nucleotide mt protein-coding gene sequences in tblastx searches of the assembly, two potential mt contigs of 12.6 and 13.1 kb (contig I and II, respectively) were identified. These two contigs shared ~800 bp of high identity sequence. Two sets of primers were designed for both contigs to amplify products in ‘inward’ and ‘outward’ directions relative to the assembled contig sequence. Amplification with the inward facing primers yielded single amplicons of the expected sizes of ~9.4 and 8.9 kb for contigs I and II, respectively (Fig. 1a). Amplification with the outward facing primers yielded single amplicons of ~9 and 7 kb for contigs I and II, respectively. The products for contig I overlapped by 222 and 351 bp. The amplicons for contig II only overlapped on one side by 1928 bp; a section of 429 bp in a region of high confidence MiSeq assembly was not originally included in the two amplifications. A third primer set for contig II was later designed to confirm the MiSeq assembly sequence in that region, with a product size of 1092 bp overlapping the larger two amplicons by 217 and 445 bp. If the two originally assembled contigs were parts of a single circle mt genome, that circle size would need to be at least ~25 kb. However, combined sizes of the inward and outward amplicons were only ~18 and 15 kb for contigs I and II, respectively, indicating they were two separate circles.

Southern blots further supported the size and existence of the two circles (Fig. 1b). When hybridized with restriction digested genomic DNA, a probe designed to hybridize to a segment of the shared sequence resulted in two visible bands of the predicted sizes for each of three different restriction enzymes. Congruently, hybridization of probes designed to sequences specific to each circle resulted in a single visible band of the predicted size for each circle and each of three different restriction enzymes. No additional bands were observed in any of the blots, even after extended exposure. Cloning and Sanger sequencing of the above described PCR products yielded the final complete circle sequences (Fig. 1c).

### Mitochondrial DNA genome structure and gene content of the two circles

The larger circle, denoted mtDNA-I (GenBank Accession No. KU726971), was 17,757 bp and contained complete open reading frames for ten proteins, *cox1-3*, *nad2-6*, *nad4l* and *cob*, as well as sequences for *trnR* and *trnT* (Fig. 1c). The smaller circle, denoted mtDNA-II (GenBank Accession No. KU726972), was 14,365 bp and contained complete open reading frames for two proteins, *atp6* and *nad1*, the 12S and 16S rRNAs (*rrnS* and *rrnL*), and the 20 remaining tRNA genes. All genes were coded on the same strand in both circles, and appropriate three dimensional structures were found for all tRNAs with the possible exception of *trnS2* (Additional file 1).

Both mt circles had non-coding sequence (ncs) between most genes, which may have impacted annotation precision. In mammals most mt genes are abutting or have just a few intergenic bases, assisting in the determination of initiation and termination codons, particularly in cases where the abbreviated stop codons T-- or TA- are used [26]. The stop codons for three *G. ellingtonae* genes (*cox1*, *cox2* and *nad1*) were not located with certainty as the open reading frame either continued beyond the conserved end of other tylenchid peptide sequences or into the next gene (Table 1). For genes with a clear stop codon, TAG was used seven times and TAA was used twice. The initiation codons TTG and TTA were used in eight and two genes, respectively, and the codons ATA and ATT were each used once. Due to open reading frames in the 5′ direction extending beyond conserved peptide start sites and multiple nearby potential initiation codons, the start codons for *cox1*, *nad1* and *nad6* are uncertain. For mtDNA-I, the ncs between genes in the non-shared region ranged from 7 to 325 bp, with an average of 109 bp for the ten intergenic ncs (Additional file 2). For the 22 ncs in mtDNA-II, nine were less than 10 bp in length (Additional file 2). There was a 670 bp sequence between *trnP* and *trnI* that contained a 366 bp *nad5* pseudogene. A 1686 bp ncs between *trnV* and *trnP* contained a 1404 bp open reading frame that yielded no hits (e <10) in a blastp search of GenBank non-redundant proteins. Clustering of tRNA genes was evident in mtDNA-II, with sections containing 8, 5, and 4 consecutive (but with intergenic ncs) tRNA genes.

**Table 1:** Start and stop codons and mtDNA gene lengths for *Globodera ellingtonae* and two tylenchid nematodes

	*Globodera ellingtonae*			*Heterodera glycines* ^a^	*Meloidogyne arenaria* ^b^
Gene	Initiation codon	Stop codon	Length (bp)	Length (bp)	Length (bp)
*atp6*	ATA	TAG	588	570	552
*cob*	TTG	TAA	1074	1073	1015
*cox1*	TTG	T*	1509	1521	1522
*cox2*	TTG	*	675	657	693
*cox3*	ATT	TAA	774	762	762
*nad1*	TTG*	*	843	834	850
*nad2*	TTG	TAG	810	804	802
*nad3*	TTG	TAG	306	334	315
*nad4*	TTG	TAG	1206	1152	1173
*nad4l*	TTA	TAG	228	222	237
*nad5*	TTG	TAA	1515	1537	1474
*nad6*	TTA*	TAG	402	439	399
*rrnL*			817	806	804
*rrnS*			674	673	610

^a^GenBank accession number HM640930

^b^GenBank accession number NC_026554

Asterisks (*) indicate uncertainties

**Fig. 2:**
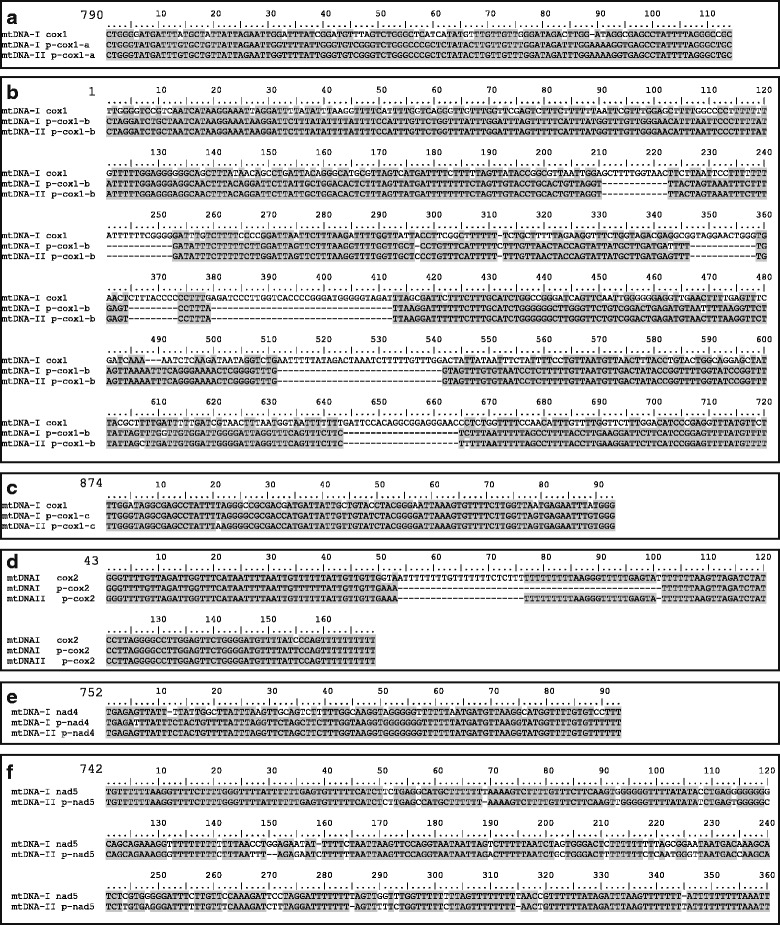
Alignments of pseudogene (ps) and functional gene sequences. Amino acid translations were used to guide alignment of the nucleotide sequences. Positions that are identical between >50 % of sequences have a *grey background*. The nucleotide of the functional gene at which the alignment begins is given at the start of each alignment. **a** *p-cox1-a*; (**b**) p-cox1-b*; (**c**) *p-cox1-c*; (**d**) *p-cox2*; (**e**) *p-nad4*; (**f**) p-nad5*. *The final 7 and 9 bp of alignments for *p-cox1-b* and *p-nad5* were removed for space considerations

The circles shared a high sequence identity ~6.5 kb region, hereafter denoted “shared region”, in their longest ncs. There were 98 % identical sites in the ~5.1 kb shared sequence region from mtDNA-II position 1398 to 6519. However, a 986 bp alignment of the start of the shared region (mtDNA-I positions 1–951; mtDNA-II positions 6–898) had higher sequence divergence, with 87 % identical sites and several short alignment gaps totaling 13 % of the aligned sequence length (Additional file 3). The alignment of sequences from mt-DNA-II positions 899 to 1173 had 99 % identity and was followed by a 224 bp gap in mtDNA-I relative to mtDNA-II positions 1174–1397. A section of sequence rich in pseudogenes occurred at mtDNA-II positions 3878 to 6115. It contained three different *cox1* pseudogenes, sizes 93, 114, and 597 bp as well as pseudogene sequences of *nad4* (94 bp) and *cox2* (121 or 145 bp for mtDNA-I or II, respectively) (Fig. 1c; Table 2). One other pseudogene (366 bp), for *nad5*, was identified only in a unique region of mtDNA-II. The pseudogenes present in the shared region were 99 to 100 % identical between the two circles, with the exception of a 24 bp deletion in the mtDNA-I *p-cox2*; however, they had much lower identity with the functional copies of the genes, from 63 to 98 % identity in non-gapped sequence (Fig. 2; Table 2). Additionally, the three *p-cox1* pseudogenes were not in consecutive order relative to their homologous positions in the functional genes (Fig. 1c; Table 2). The total ncs of each circle had lower AT content than the coding sequence, with 62.5 and 63.9 % AT in the ncs versus 71.0 and 76.9 % AT for the coding sequences of mtDNA-I and mtDNA-II, respectively (Table 3). In total, mtDNA-I had 8452 bp (47.6 %) ncs, 696 bp (3.9 %) pseudogene sequence, and 8610 bp (48.4 %) functional gene sequence; mtDNA-II had 9259 bp (64.5 %) ncs, 1085 bp (7.6 %) pseudogene sequence, and 4018 bp (28.0 %) functional gene sequence.

**Table 2:** Characteristics of *Globodera ellingtonae* mitochondrial pseudogenes

				mtDNA-I-mtDNA-II		Pseudo-functional	
Pseudogene	Functional gene start nt^a^	Pseudogene length^b^	Functional gene span^b^	% identity^c^	% gaps^d^	% identity^c^	% gaps^d^
*p-cox1-a*	790	114	113	100	0	81	1
*p-cox1-b*	1	597	723	99	0	63	18
*p-cox1-c*	874	93	93	99	0	91,90^e^	0
*p-cox2*	44	121,145^e^	169	100	17	98	28,14^e^
*p-nad4*	752	92	93	99	0	80	1
*p-nad5*	742	366	369	-	-	89	2

^a^The position given is the nucleotide of the functional gene at which the pseudogene sequence begins. ^b^Due to gaps in the sequences, the length of the pseudogenes and the span of the functional gene with which they align are both listed. ^c^Percent sequence identities are for non-gapped aligned sequence. ^d^Percent gaps are for the entire length of the alignment. ^e^Where two values are separated by a comma, the first value is for mtDNA-I and the second for mtDNA-II

**Table 3:** *Globodera ellingtonae* mitochondrial DNA characteristics compared with other tylenchid nematodes

	Circle	Longest non-coding^a^		Total non-coding^b^		Coding^c^
Species	size	bp	%AT	bp	%AT	%AT
*Globodera ellingtonae* mtDNA-I	17.8 kb	8059	62.0	9147	62.5	71.0
*Globodera ellingtonae* mtDNA-II	14.4 kb	7232	62.3	10,347	63.9	76.9
*Radopholus similis* NC_013253^d^	16.8 kb	3705	87.3	3984	87.4	84.8
*Pratylenchus vulnus* NC_020434	21.7 kb	6858	73.0	8821	72.7	74.6
*Meloidogyne chitwoodi* NC_024096	18.2 kb	5404	86.1	5896	82.3	83.5
*Meloidogyne incognita* NC_024097	17.7 kb	4097	80.8	5311	81.4	84.3

^a^The longest single stretch of non-coding sequence

^b^All non-coding sequence combined

^c^ The combined 36 mt genes

^d^GenBank accession numbers follow species names

### Gene order conservation with relatives

**Fig. 3:**
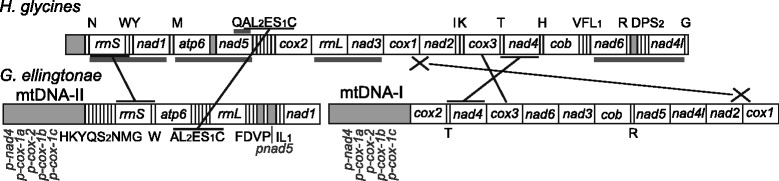
Comparison of *Globodera ellingtonae* and *Heterodera glycines* mt genome organization. Gene order is shown, with protein-coding and rRNA gene names inside boxes (*boxes* not to scale) and tRNA gene abbreviations and pseudogene names given outside the boxes. Grey boxes represent non-coding sequence (ncs). *Grey bars* under *H. glycines* (Accession #HM640930) genes indicate gene order conservation between *H. glycines*, *Radopholus similis*, *Pratylenchus vulnus*, *Meloidogyne chitwoodi*, and *M. incognita*, not necessarily including intermediate ncs or tRNA genes. *Black lines* indicate genes adjacent in both *G. ellingtonae* and *H. glycines*

The gene order in the circles of *G. ellingtonae* shows little conservation with that of tylenchid relatives outside the genus. We omit gene order comparisons within the genus *Globodera* as the mt genomes of both *G. pallida* and *G. rostochiensis* are undergoing considerable revision (W Phillips, S Eves-van den Akker, and V Blok, unpublished). Five pairs of genes consistently adjacent to each other in the currently available mt genomes of *Heterodera*, *Radopholus*, *Pratylenchus*, and *Meloidogyn*e are not adjacent in *G. ellingtonae* (Fig. 3). To the contrary, there is a series of five tRNA genes whose order is conserved between *G. ellingtonae* and *H. glycines* but not with the other genera. An additional curiosity is the reversal of order of two pairs of adjacent genes (*cox1* and *nad2*, and *cox3* and *nad4*) in *G. ellingtonae* mtDNA-I compared to *H. glycines*. Partial mt genome sequences are available for *H. cardiolata* and *Punctodera chalcoensis*, spanning six and seven protein-coding genes, respectively. The partial mt sequences of these two species have 100 % synteny with *H. glycines*, exhibiting the same relative reversal compared with *G. ellingtonae* of *cox1* and *nad2* and *cox3* and *nad4*.

### Differential copy numbers of the two mtDNA circles

**Fig. 4:**
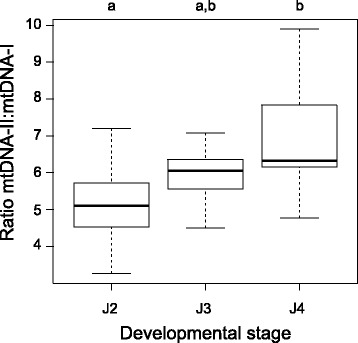
Ratio of mtDNA-II:mtDNA-I copy number in individual nematodes. Absolute copy numbers of each mtDNA circle were determined by qPCR using individual nematodes of different developmental stages, and the ratio of mtDNA-II to mtDNA-I copy number was calculated. The bold bars within the boxes indicate sample means, the edges of the boxes indicate first and third quantiles, and the whisker bars indicate minimum and maximum values (J2: *n* = 10, J3: *n* = 8 and J4: *n* = 8)

Differential copy numbers of the two mt circles was supported by three different methods of quantification. Densitometry of Southern hybridization bands in the blot probed by shared sequence indicated bands of mtDNA-II were stronger than mtDNA-I, with a ratio of 15.4:1 for the PacI digest and 3.6:1 for the EcoRI digest (Fig. 1b). The mapping of MiSeq reads to unique sections of each circle yielded a mean coverage of 787× for mtDNA-II and 149× for mtDNA-I, a 5.3:1 ratio. To test whether there was a similar ratio at the individual level and whether the ratio changed with developmental stage, we performed quantitative PCR (qPCR) on individual nematodes. There was a pattern of increasing ratio of mtDNA-II:mtDNA-I copies as nematodes progressed to later developmental stages (Fig. 4). Although there was wide variation within individual stages, a TukeyHSD test indicated that individual fourth-stage juveniles (J4) had a significantly higher mtDNA-II:mtDNA-I copy ratio than second-stage juveniles (J2), mean 6.91 ± 0.56 s.e. vs. 5.10 ± 0.35 (*p* = 0.01), while the ratio for third-stage juveniles (J3) ratio was intermediate between the two, 5.94 ± 0.27.

## Discussion

### Mt genomes in nematodes

**Fig. 5:**
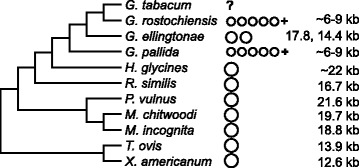
Mitochodrial genome characteristics of select tylenchid nematodes. The number and size of circles in the genomes of tylenchid species closely related to *Globodera ellingtonae* with outgroups (*Trichuris ovis* and *Xiphenema americanum*) are shown. The + following *G. rostochiensis* and *G. pallida* circles indicates that each has at least 5, but potentially more circles. A schematic of well-supported relationships among the species is shown based on previously published phylogenies [2, 29]

Our discovery and description of the mt genome of *G. ellingtonae*, composed of two large circles, provides new insight into a phenomenon rare in the metazoa: the division of mt genomes into multiple circles. The context of this discovery is especially intriguing as *G. ellingtonae* is phylogenetically intermediate between *G. pallida* and *G. rostochiensis*, both of which have at least five mini-circles, ranging in size from ~6 to 9 kb (Fig. 5) [19, 20, 25]. At the structural level, the finding of species with such different circle numbers and sizes is a pattern as yet unseen within any other metazoan genera. Of interest is the as yet unknown karyotype of the unsequenced mt genome of the tobacco cyst nematode, *G. tabacum*, sister to *G. rostochiensis*. There are very few complete mt genomes available for nematodes closely related to the *Globodera*, i.e. in the monophyletic clade of the infraorder Tylenchomorpha that includes *Globodera*. There are complete mt genomes available for *Radopholus similis* and *Pratylenchus vulnus* [27, 28] showing single circles in these species. Recent comparative studies have yielded single circle mt genomes for five species of *Meloidogyn*e [29, 30]. There is one nearly complete *Heterodera* mt genome available (*H. glycines*), missing a section of presumable ncs, but PCR evidence indicates it too is a single circle mt genome [31]. There is PCR evidence of a mini-circle containing only a subset of mt genes in a close *Globodera* relative, *Punctodera chalcoensis*, indicating it likely has a multipartite genome; however, the partial mt sequence available for it has 100 % synteny with the mt genome of *H. glycines* [31]. Further investigation of the mt genome karyotype in *P. chalcoensis* and other basal relatives within the genus *Globodera* will be key in determining when the multipartite condition in this lineage first formed.

The single mt circles in these tylenchid relatives are among the largest mt genomes reported in nematodes, which most commonly are under 16 kb, defying the usual conservation of mt genome compactness in the metazoa (Table 3; Fig. 5). As mt genome size increases in these tylenchids so does the length of their ncs, with *P. vulnus* having the largest circle, 21.7 kb, and longest ncs, 6.8 kb. Yet the two circles of *G. ellingtonae*, individually smaller than the mt genome of *P. vulnus*, have even longer single stretches of ncs, 7.2 and 8.1 kb. Despite the exceptionally long stretches of ncs in tylenchid nematodes, no high identity sequence regions (i.e. longer than 20 bp at >90 % identity or longer than 40 bp at >80 % identity) were found between the ncs of *G. ellingtonae* and that of *P. vulnus*, *R. similis*, or any of the five sequenced species of *Meloidogyne* [27–30]. A highly unusual feature of the ncs in *G. ellingtonae* is its reduced percent AT composition compared to its own coding sequence and to the ncs of its tylenchid relatives: ~62 % AT in the *G. ellingtonae* ncs as compared to 73 to 87 % in the other genera. Nowhere in the ncs was there a long stretch of extremely high AT content as seen in the model nematode *C. elegans*. This is of particular note as “AT-rich region” is often used as a synonymous term for the non-coding region and/or control region in mammalian and other mt genomes.

### Functional convergence in multipartite mt genomes

Given that a multipartite genome organization has appeared independently in such disparate metazoan taxa as rotifers, nematodes, and insects, the question arises of whether this state is mildly deleterious, neutral, or of benefit. A possible functional advantage of having the mt genome in multiple circles is the localization of genes to different transcriptional units. Ojala et al. [32] proposed that human mtDNA is transcribed as a single polycistronic molecule, which is then further processed to produce separate tRNAs, rRNAs, and mRNAs for individual genes. Although others have shown that transcription may have multiple sites of origin and termination, the transcripts are generally polycistronic [26]. It therefore seems advantageous for genes coding for proteins for the same ETC enzyme complex to be on the same transcriptional unit, yielding more efficient co-regulation of their expression [33]. By dividing the genome into separate circles, separate transcriptional units are created. The most extreme example of this is observed in several species of blood-sucking lice for which each protein-coding gene is on a separate circle [14].

Consistent with this model of segregation of transcriptional units, gene types were highly partitioned between the two *G. ellingtonae* mt circles, with 10 of 12 protein-coding genes on mtDNA-I and both rRNA genes, *atp6* and *nad1*, and 20 of 22 tRNA genes on mtDNA-II (Fig. 1c). In two other metazoan genera reported to have a mt genome split into two similarly sized circles, *Liposcelis* and *Brachionus*, gene types (protein-coding, rRNA, or tRNA) also are distributed unevenly between the two circles, although not to the same extreme. The *Brachionus* mt genomes, which are structurally similar in the two species with complete sequences, have moderately segregated distribution of gene types, with the mtDNA-I having four protein-coding (*atp6*, *cob*, *nad1*, and *nad2*), both rRNA, and 14 tRNA genes, and mtDNA-II having eight protein-coding and nine tRNA genes [11, 34]. The mt genes of *L. paeta* and *L. entomophila* exhibit greater segregation, although the full complement of 37 mt genes were not all identified in either species [35]. Chromosome I of *L. paeta* has ten protein-coding, one rRNA, and three tRNA genes, while chromosome II has three protein-coding, one rRNA, and 11 tRNA genes. Chromosome I of *L. entomophila* has 11 protein-coding but no rRNA or tRNA genes; while chromosome II has one protein-coding, both rRNA, and 14 tRNA genes. The gene types are more evenly distributed in *L. bostrychophila*, with seven protein-coding, one rRNA, and 14 tRNA genes on chromosome I, and six protein-coding, one rRNA, and nine tRNA genes on chromosome II [12]. Nonetheless, potential bias in the genes for the different ETC complexes is evident, as chromosome II contains both *atp* genes and four *nad* genes, whereas chromosome I has all *cox* genes, *cob* and the other three *nad* genes. The convergent characteristic of gene type segregation between the two circles in these three very disparate genera is consistent with the hypothesis of a functional benefit to such an organization. Although the mt genome of the nematode *Rhabditophanes* sp. KR3021 is divided into two circles, the gene order is highly conserved with the conventional order found in several genera of related nematodes including *Bursaphalenchus*, *Caenorhabditis*, and *Ascaris*, suggesting either recent genesis of the multipartite structure or strong selection pressure for conservation in that lineage [13].

Differential copy numbers of each circle found in two-circle genomes further supports a possible functional role for a multipartite structure. That mtDNA-II of *G. ellingtonae* is in higher relative copy number may relate to a greater requirement for the “building blocks” (tRNAs and rRNAs) of the protein-coding genes than for the mRNA templates of those proteins. Differential copy numbers were found in *Liposcelis* and *Brachionus* as well; in *B. plicatilis* there was a 4:1 ratio of mtDNA-I to mtDNA-II and in *L. bostrychophila* mt chromosome I was twice as numerous as mt chromosome II. Although the differentiation in gene composition of those mt circles was not as great as in *G. ellingtonae*, the commonality among all three taxa is that the higher copy number circle contained more tRNA genes. Additionally, for both *G. ellingtonae* and *Brachionus* the higher copy circle contained *atp6*, *nad1*, and both rRNA genes. It is uncertain whether the increasing mt copy differential corresponding to developmental stage in *G. ellingtonae* serves a direct function or is a byproduct of some other force. For example, one functional explanation is that increased replication is associated with increased transcription of mtDNA-II. However, a possible non-functional explanation is that smaller mt circles may have a replicative advantage, resulting in differential copy numbers [36]; in other words, mtDNA-II may be at higher copy number as a byproduct of a “selfish” DNA process. The circle size and copy ratio relationship in *Brachionus* fits such a model, but in *L. bostrychophila* it is the slightly larger circle that has the higher copy number. Experiments with the nematode *C. elegans* indicate that the majority of its mt genome replication occurs in the gonads, with much of the mtDNA of somatic cells simply derived from mtDNA dispersed during embryonic development [37]. If this is also the case in *G. ellingtonae*, just a small increase in replication or slightly lower degradation rate of mtDNA-II in somatic tissue could generate the mtDNA-II:mtDNA-I ratio increase with age.

### Formation of multipartite mt genomes

The mechanism by which the multipartite genome in *Globodera* and other lineages arose is uncertain. Movement of stretches of mtDNA can occur by various mechanisms including slipped-strand mispairing and recombination, resulting in rearrangements such as duplications, deletions, and inversions [38]. If rearrangements exist in a subset of the mtDNA creating a heteroplasmic population of mtDNA, further differentiation of the two (or more) types of mtDNA could eventually lead to circles with distinct gene structure and/or segregated gene content. Some have proposed multipartite mt genome formation via tandem duplication followed by random loss [35]. A particularly rapid path to such differentiation would be an initial duplication of the entire genome followed by degradation of distinct gene sets on or recombination between initially heteroplasmic circles. Evidence was recently reported supporting mtDNA replication by a rolling circle mechanism in *C. elegans* [6]. If rolling circle replication of mtDNA is conserved among nematodes, an ancestral duplication of the mt genome in the *Globodera* lineage is easily envisioned. Whole genome duplication followed by gene loss has resulted in higher rates of evolution in yeast [39]. Following such duplication, selection pressure could favor maintaining operational copies of gene functional groups on the same transcriptional units and thus causing segregation between circles. Given the unusual pattern of mt karyotypes in the three sequenced *Globodera* species, it is unclear whether the formation of the ancestral multipartite structure involved a single split into two circles or a “shattering” of the genome into multiple circles that were subsequently re-joined to form one or both large circles of *G. ellingtonae*. Future investigation of potentially multipartite mt genomes of nematode species basal to this group is needed.

As remnants of reorganization, the structure of the *G. ellingtonae* pseudogenes may provide insight into the etiology of the divided genome. Curiously, the pseudogenes of each circle are more similar to each other than they are to the respective functional gene. Three different hypotheses could explain this pattern. 1) All the pseudogenes were created when there was still a single (perhaps duplicated) circle, they differentiated from the functional genes, and then they were copied into both circles when the circles formed. 2) Two circles were formed and only one had the pseudogenes or different pseudogene segments were on different circles. Following pseudogene differentiation from functional sequence, recombination resulted in duplication of the pseudogene(s) into the other circle. 3) The process by which the two circles formed resulted in duplicate pseudogenes on both circles that were then subject to strong homogenization pressure. Differences in the divergence of the pseudogenes provide other clues. The longest evident pseudogene, *p-cox1-b*, is also the most differentiated from the functional gene, with both high deletion levels and high sequence divergence in the ungapped sequence. Preceding it in the ncs is *p-cox2*, which is the only pseudogene with a deletion between the copies on the different circles. This suggests the possibility that the ancestral creation of this *p-cox2* to *p-cox1-b* region yielded a recombination hot spot, creating the peripheral more identical pseudogenes during recombination events while facilitating rearrangement of the genome. Such analysis of pseudogene structure may be hindered by the incomplete detection of highly degraded pseudogenes. A high rate of pseudogenization is also found in *Liposcelis*, with four, eight, and 15 pseudogenes in *L. bostrychophila*, *L. paeta* and *L. entomophila*, respectively, while no pseudogenes were reported in *Brachionus*.

The 2.2 kb of sequential pseudogenes in the mtDNA of *G. ellingtonae* is a subset of the 5.1 kb stretch of sequence with 98 % identity between the two circles. We did not identify a functional control region on the circles, although it is most likely located either in the 5.1 kb region with 98 % pairwise identity or in the upstream 1 kb sequence with 87 % pairwise identity. The control region presumably would have been necessary in both/all progenitors of the current two circles for their continued replication. However, if the control region is not in the 98 % identity region, then the latter may have been recently derived from a ncs region that diverged in just one of the circles but then was duplicated into the second circle. If that sequence region was always on both circles, its high conservation could be explained either by concerted evolution creating homogenization of the ncs on both circles, as seen in the two ncs regions in some snakes [40], or by the two-circle state being very recently derived and rates of evolution much higher in the protein-coding region than in the large ncs.

There are intriguing similarities and differences in the distribution of ncs in the other genera with two-circle multipartite genomes. Both *G. ellingtonae* and *Brachionus* spp. have long stretches of highly similar ncs shared between circles, ~6.5 and 4.9 kb, respectively [11]. However derived, long stretches of homologous sequence between otherwise different circles provides a region of increased recombinational potential. Although the mt genomes of both *L. paeta* and *L. entomophila* have a higher than usual proportion of ncs, it is highly dispersed between coding genes, particularly between tRNA genes; it is very unequally distributed between circles with much more on chromosome II; and little ncs is shared between circles, with just three stretches of shared sequence, each less than 400 bp [35].

### Maintenance and inheritance of multipartite mt genomes

Once differentiated mt circles have formed, regardless of the cause, organisms must have a mechanism to maintain function of all circles to preserve mitochondrial function. To date, there has been little discussion in the literature of how multipartite genomes are partitioned in nucleoids. Generally, mtDNA is packaged with proteins into nucleoids, each containing from one to ten copies of the mt genome, that are evenly distributed on the mitochondrial inner membrane [41, 42]. An interesting line of inquiry is whether all circles of a multipartite genome are contained within individual nucleoids, and if so whether the copy ratio of the different circles is consistent between nucleoids. In single circle genomes with heteroplasmic variants, there is evidence both for random distribution of heteroplasmic types and for within-nucleoid purifying selection [41, 43]. Additionally, the mechanism by which the full complement of circles of a multipartite genome is faithfully transmitted to the next generation is unknown, but certainly involves nucleoid organization. It is of note that in the case of *G. ellingtonae*, where the circle copy number ratio changes with development, there must exist a mechanism by which the ratio is maintained within the germ line or reset during gametogenesis or embryogenesis. The multipartite mt genome in *G. ellingtonae* could provide a model system for deconstructing mechanisms of nucleoid based regulation of mtDNA both in somatic cells and during germline development.

## Conclusions

The complete mt genome of the nematode *G. ellingtonae* is unique and distinct from other multipartite mt genomes. Its circles are larger and show more segregation of gene repertoire than any other multipartite metazoan mt genome recorded to date. Its structure is particularly interesting in the context of the mt genome structure of its sibling species, *G. pallida* and *G. rostochiensis*, which consist of several much smaller circles. This is the only metazoan genus reported to contain species with both two circle and several circle genomes. It is likely the several pseudogenes and the large shared non-coding region are factors in the rapid evolution of these circles. Differential copy numbers of the mt circles, with the more abundant circle encoding rRNA genes and the majority of tRNA genes, is congruent with a model of functional differentiation of the circles. The change in the copy number ratio of the two circles as nematode development progresses and its reset in the next generation provides a potentially rewarding experimental system for studying mtDNA regulation.

## Methods

### DNA extraction and sequencing

Cysts of *G. ellingtonae* were collected from the field in which it was discovered at Powell Butte, OR. Complete genomic DNA was extracted from a pool of ~30,000 newly hatched second-stage juveniles (J2) using a Qiagen DNeasy Blood & Tissue kit. The nematodes were ground in lysis buffer in a 1.7 ml centrifuge tube with a plastic pestle and incubated in the proteinase K solution at 56 °C for 2 h. The resulting DNA was sheared with a Diagenode Bioruptor Pico for 50 s. A sequencing library was constructed using an Illumina TruSeq DNA Kit, with fragmented ligated targets of ~650–750 bp gel-excised following adapter ligation. Sequencing was performed using 1/3 of a lane in the Illumina MiSeq system for 301 × 2 cycles (paired-end). Illumina MiSeq reads were de novo assembled in CLC Genomic Workbench (CLC Bio-Qiagen) using the following parameters: bubble size = 294, word size = 64, minimum contig length = 600, mismatch cost = 2, insertion cost = 2, deletion cost = 2, length fraction = 0.7, similarity fraction = 0.8. To find potential mtDNA, nucleotide sequences of *Heterodera glycines* (GenBank Accession #HM640930) and *G. pallida* (GenBank Accession #AJ249395) mtDNA protein-coding genes were used as queries in a tblastx search of the subset of assembled contigs >2 kb in length and with >5× the average coverage to avoid nuclear mitochondrial DNA sequences (numts).

Two assembled contig sequences were identified as containing mtDNA sequences. They were 12.6 and 13.1 kb (henceforth denoted mtDNA-I and mtDNA-II, respectively) and contained ~800 bp of high identity sequence. Primers were designed to unique sequences of each contig in order to PCR amplify, clone, sequence and confirm circularity of each mtDNA piece (Additional file 4). Each circle was PCR amplified in large overlapping segments: mtDNA-I in two segments that were 9388 and 8944 bp; and mtDNA-II in three segments that were 8884, 6982 and 1092 bp. Thermo Scientific Phusion Flash High-Fidelity PCR mix was used for amplification with the following thermal cycling parameters: an initial denaturation at 98 °C for 10 s followed by 37 cycles of 98 °C for 1 s, 58 °C for 5 s, and 72 °C for 2 min 45 s, with the last 72 °C extension for 5 min. To prepare amplification products for TA cloning, they were first purified with a DNA Clean & Concentrator-5 mini-column (Zymo Research). Promega Go-Taq DNA polymerase (2u/reaction) was used to add an A-overhang to the 3′ ends of the DNA by incubating the purified product in 1× GoTaq Buffer, 2.5 mM MgCl_2_, and 0.2 mM dATP at 72 °C for 20 min. The A-tailed products were then run on a 0.8 % agarose, 1× TAE gel containing crystal violet and the appropriate sized bands were cut from the gel and extracted with S.N.A.P. purification columns (Thermo Scientific). To further concentrate the gel extracted DNA, it was again passed through a DNA Clean & Concentrator-5 mini-column. It was then ligated into the pCR-XL-TOPO vector (Invitrogen), which was subsequently used to transform One Shot chemically competent TOP10 *E. coli* (Invitrogen). Plasmid DNA was purified using the QIAprep Spin Miniprep Kit (Qiagen) and sequenced with BigDye Terminator v3.1 Cycle Sequencing Kit (Applied Biosystems) reactions run on an ABI 3730 capillary sequence machine. All mtDNA sequence segments were covered by at least two clones, or more when needed to resolve discrepancies.

### Annotation of mtDNA and sequence analysis

Mitochondrial genes were identified through a combination of methods. Sequences were analyzed with MITOS [44] to identify protein-coding, tRNA and rRNA genes. Additionally, to identify protein-coding genes and pseudogenes, all open reading frames greater than 80 nucleotides in length were translated and analyzed for homologous sequences with a blastp search of GenBank non-redundant proteins. For protein-coding genes, assignment of start and stop sites was guided by alignment of translated open reading frames with mt peptide sequences from the other tylenchid nematodes *G. pallida*, *H. glycines*, *Meloidogyne sp.*, *Radopholus similis*, and *Pratylenchus vulnus* (accession numbers Table 3 and above). The start and stop of the two rRNAs also was based on alignment to the same set of species. Three programs were used to find tRNA genes: MITOS, tRNA-scan, and Arwen [44–46], with the highest scoring tRNA predictions used for the final annotation. Annotations from MITOS were manually adjusted, sequences were aligned, and maps created using the Geneious software. Final figures were created using Adobe Illustrator, with alignment figures initially created using Bioedit [47]. Statistical tests were performed and the qPCR figure were created using the package R [48].

### Southern hybridization

As a secondary confirmation of circle sizes and configurations, Southern hybridization analysis was performed using the Pierce North2South Labeling and Detection kit (Thermo Scientific). Restriction enzymes and probe locations that would generate informative Southern blot bands were chosen based on restriction enzyme mapping of the DNA sequences. A 422 bp probe specific to mtDNA-I, a 622 bp probe specific to mtDNA-II, and a 875 bp probe designed to shared sequence were generated by biotin-II-dUTP random prime labeling using Klenow DNA polymerase (probe locations shown in Fig. 1c). Double-stranded DNA for the labeling reaction was generated by PCR using plasmid DNA containing the appropriate cloned mtDNA fragments as template (primers sequences in Additional file 4). PCR products were treated with ExoSAP-IT (Affymetrix) before Klenow labeling. Before use, the probes were precipitated in ethanol with ammonium acetate and glycogen.

Total genomic DNA was extracted from a pool of ~90,000 hatched J2 nematodes. Nematodes were incubated in extraction buffer (0.1 M NaCl, 10 mM Tris pH 8, 10 mM EDTA, 1 % SDS, 1 % beta-mercaptoethanol, 100 μg/ml proteinase K) at 60 °C for 3 h, followed by two phenol:chloroform extractions and one chloroform extraction. DNA was precipitated with two volumes 100 % ethanol containing a final NaCl concentration of 0.2 M. The resulting total genomic DNA (6 μg DNA/reaction) was digested with three different restriction enzymes: EcoRI, KpnI, and PacI. Using one third of each reaction per lane, the products from the three separate enzymes were loaded side by side in three sets alongside a non-labeled 1 kb DNA ladder on a single 0.8 % agarose gel.

The DNA was transferred to a single positively charged nylon membrane overnight using capillary transfer and then UV crosslinked for 3 min at 120 mJ. The membrane was then cut to separate the three digest sets. Each membrane piece was hybridized with a probe overnight at 55 °C in hybridization buffer containing 100 ug/ml salmon sperm DNA. Following manufacturer’s directions for the North2South Detection kit, membranes were washed, incubated with streptavidin-HRP followed by the chemiluminescent substrate, and exposed to X-ray film for various exposure times ranging from 30 s to 32 min. To compare band intensities from the two circles, blot images were photographed using GeneSys software (Syngene) and GeneTools was used for densitometry analysis of background corrected bands.

### Read coverage

Bowtie2 in local mode was used to map 10,182,218 Illumina MiSeq reads (unpaired, mean length 280 bp) back to unique sections of each mtDNA: nucleotides (nt) 8795 to 14,993 for mtDNA-I and nt 6526 to 11,726 for mtDNA-II [49]. To reduce edge effects, average read coverage was determined for an internal 4460 bp region that had the most uniform coverage: nt 8985 to 13,445 for mtDNA-I and nt 6671 to 11,131 for mtDNA-II.

### Quantitative PCR

Relative copy numbers of the two mtDNA circles were determined in individual nematodes at three different stages of development using quantitative PCR (qPCR). To obtain nematodes, cysts were incubated in 10 % potato root exudate to promote hatching of eggs. Hatched J2 were collected and applied to potato roots buried in a small layer of moistened sand. After an overnight incubation, infected roots were thoroughly washed to obtain a synchronized cohort of developing nematodes. Plants were then transplanted to Deep Rootrainers (Haxnicks), to allow easy subsequent access to the roots. Two days after the roots were washed, a subset of the roots was removed and vigorously washed with water, and then J2 nematodes were collected from macerated roots. Similarly, third- (J3) and fourth-stage juveniles (J4) were collected from macerated roots, both at 19 days post infection. All nematodes were cut in half with a micro-scalpel before placement in a DNA extraction solution [50]. The nematode-DNA extraction solution was stored at −80 °C until subsequent processing. An additional 2 μl of 2 mg/ml proteinase K was added to each sample, and the mixture was incubated at 60 °C for 1 h, followed by a 15 min incubation at 80 °C to inactivate the proteinase K. The DNA solutions were further diluted 1:25 with water, which was determined to yield optimal concentrations of template for qPCR.

Templates for standard curves were constructed from plasmid DNA containing the appropriate mtDNA fragments. Initial plasmid DNA concentration was assayed using the Qubit dsDNA BR Assay Kit and a Qubit Fluorometer (Thermo Scientific), and molarity of individual plasmid DNAs was calculated from the DNA concentration and total plasmid size. Plasmid DNA for each circle was then diluted to create standard solutions containing between 30 and 26,000 molecules/μl. From a starting concentration of 21,500 copies/μl for mtDNA-I and 26,000 copies/μl for mtDNA-II, samples were serially diluted in a 1:5 ratio. Primers were designed to specifically amplify a 170 bp fragment of mtDNA-I and a 186 bp fragment of mtDNA-II (Additional file 4). Two μl of DNA template (nematode or standard) was used in 25 μl reactions containing 1× Power SYBR Green Master Mix (Applied Biosytems) and 0.2 μM primers. All DNA samples (J2 = 10, J3 = 8, J4 = 8 individuals) for each primer set were run in triplicate in a single 96 well plate. An ABI SetpOnePlus Real-Time PCR machine (Applied Biosystems) was used for the qPCR with cycling parameters of a 10 min incubation at 95 °C followed by 40 cycles of 95 °C for 15 s and 60 °C for 60 s and then a final melt curve analysis. StepOne software was used for analysis.

## Additional files

Additional file 1: Putative secondary structures of *Globodera ellingtonae* tRNAs. (PDF 125 kb) Additional file 2: Organization of *G. ellingtonae* mtDNA-I and mtDNA-II. (DOCX 20 kb) Additional file 3: Alignment of the start of the shared sequence region between *Globodera ellingtonae* mtDNA-I and mtDNA-II. (PDF 105 kb) Additional file 4: Primer sequences. (DOCX 18 kb)

## Abbreviations

ETC: Electron transport chain
J2, J3, and J4: Second-, third-, and fourth-stage juvenile
mt: Mitochondrial
ncs: Non-coding sequence
PCN: Potato cyst nematode
qPCR: Quantitative polymerase chain reaction
