# Supplementary figures and images for: The mitochondrial genome of Globodera ellingtonae is composed of two circles with segregated gene content and differential copy numbers
Source: BMC Genomics. 2016 Sep 5;17(1):706. doi: 10.1186/s12864-016-3047-x (PMC5011991; doi:10.1186/s12864-016-3047-x)

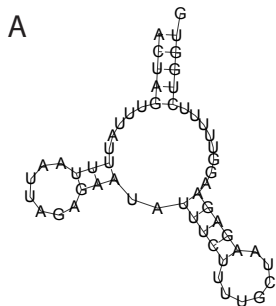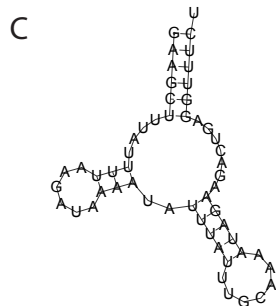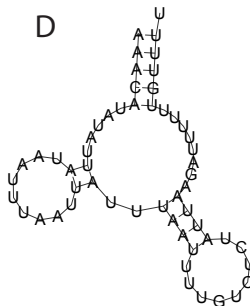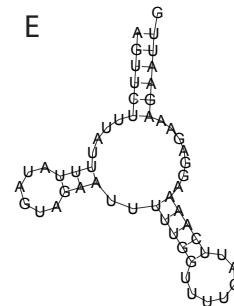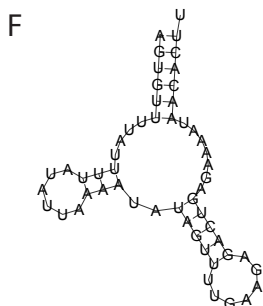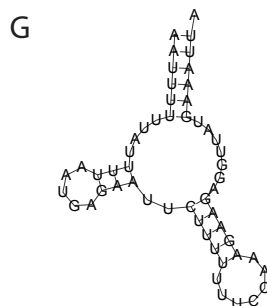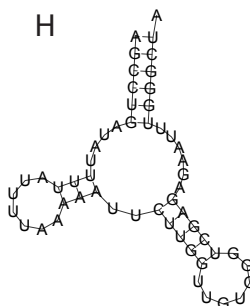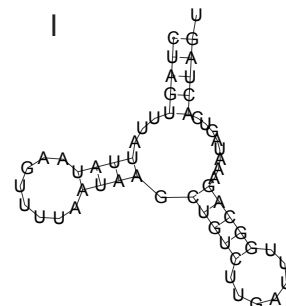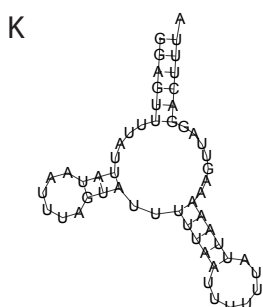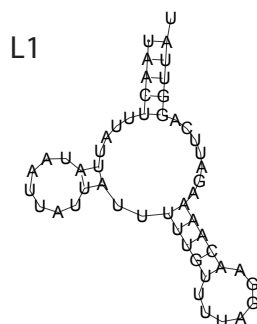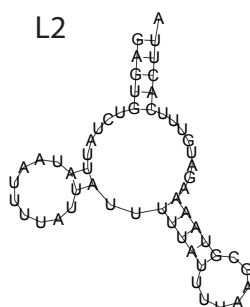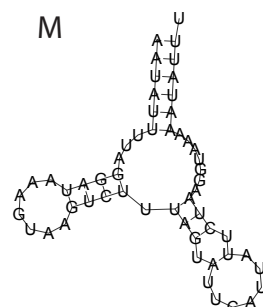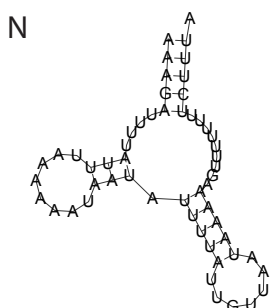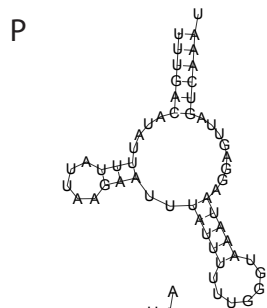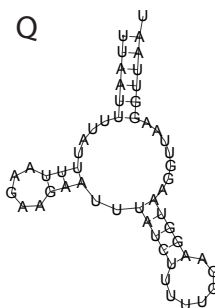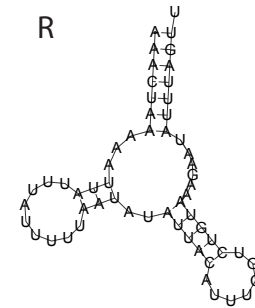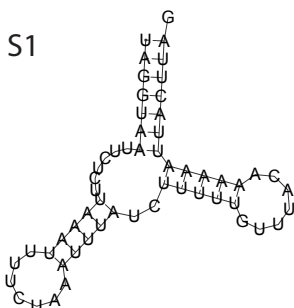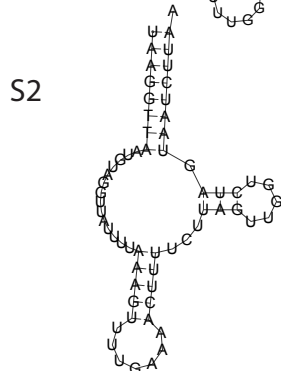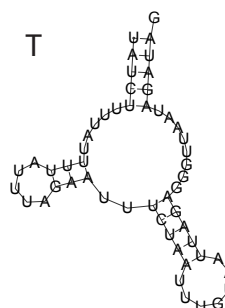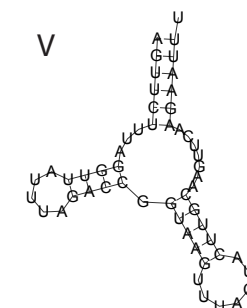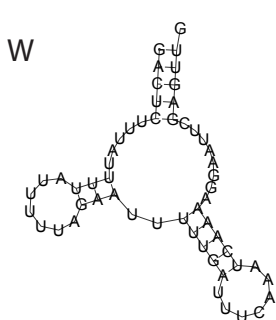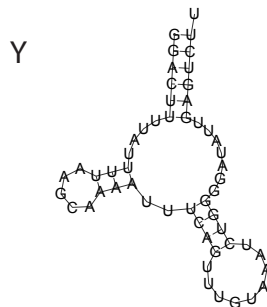

\*S2 alternate conformation

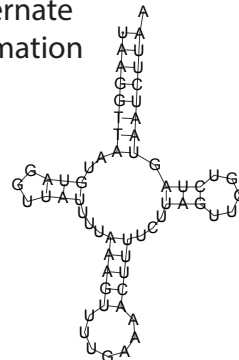

Supplement: Additional file 1: — Putative secondary structures of Globodera ellingtonae tRNAs. (PDF 125 kb) [file 12864_2016_3047_MOESM1_ESM.pdf]

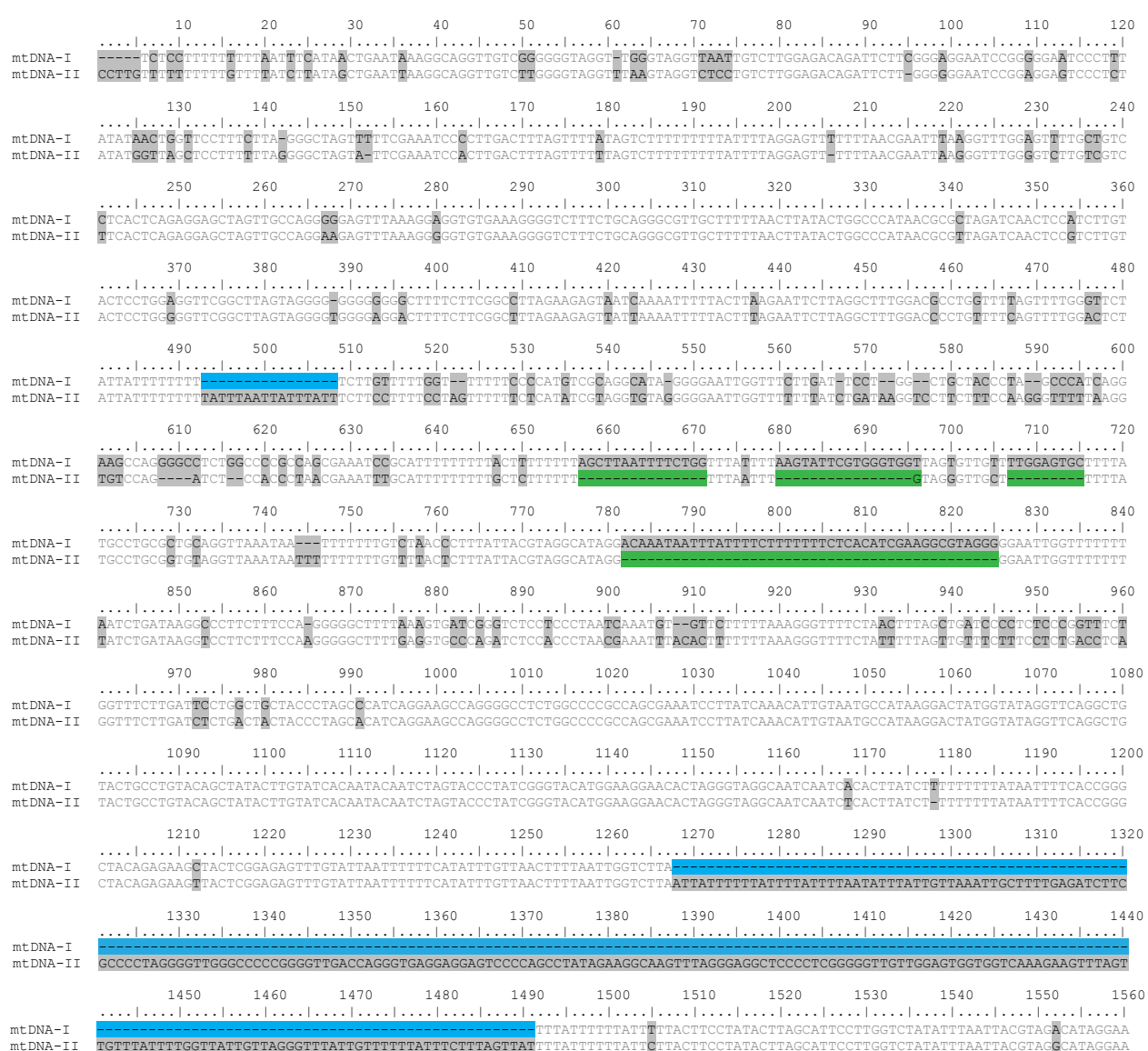

Supplement: Additional file 3: — Alignment of the start of the shared sequence region between Globodera ellingtonae mtDNA-I and mtDNA-II. (PDF 105 kb) [file 12864_2016_3047_MOESM3_ESM.pdf]
