# Supplementary material for: The mitochondrial genome of Globodera ellingtonae is composed of two circles with segregated gene content and differential copy numbers
Source: BMC Genomics. 2016 Sep 5;17(1):706. doi: 10.1186/s12864-016-3047-x (PMC5011991; doi:10.1186/s12864-016-3047-x)
Supplement: Additional file 2: — Organization of G. ellingtonae mtDNA-I and mtDNA-II. (DOCX 20 kb) [file 12864_2016_3047_MOESM2_ESM.docx]

Organization of *Globodera ellingtonae* mtDNA-I

| Name | Minimum | Maximum | Length | intergenic space following (bp) |
| --- | --- | --- | --- | --- |
| Shared sequence | 1 | 6,310 | 6,310 |  |
| p-nad4 | 3,710 | 3,802 | 93 | 15 |
| p-cox1-a | 3,818 | 3,931 | 114 | 87 |
| p-cox2 | 4,019 | 4,141 | 123 | 483 |
| p-cox1-b | 4,625 | 5,221 | 597 | 679 |
| p-cox1-c | 5,801 | 5,893 | 93 | 958 |
| cox2 | 6,852 | 7,526 | 675 | -1 |
| T | 7,526 | 7,579 | 54 | 7 |
| nad4 | 7,587 | 8,792 | 1,206 | 88 |
| cox3 | 8,881 | 9,654 | 774 | 60 |
| nad6 | 9,715 | 10,116 | 402 | 110 |
| nad3 | 10,227 | 10,532 | 306 | 269 |
| cob | 10,802 | 11,875 | 1,074 | 90 |
| R | 11,966 | 12,023 | 58 | 55 |
| nad5 | 12,079 | 13,593 | 1,515 | 46 |
| nad4l | 13,640 | 13,867 | 228 | 38 |
| nad2 | 13,906 | 14,715 | 810 | 325 |
| cox1 | 15,041 | 16,549 | 1,509 | 4,917 |

Organization of *Globodera ellingtonae* mtDNA-II

| Name | Minimum | Maximum | Length | intergenic space following (bp) |
| --- | --- | --- | --- | --- |
| Shared sequence | 6 | 6,532 | 6,527 |  |
| p-nad4 | 3,878 | 3,970 | 93 | 14 |
| p-cox1-a | 3,985 | 4,098 | 114 | 87 |
| p-cox2 | 4,186 | 4,332 | 147 | 480 |
| p-cox1-b | 4,813 | 5,409 | 597 | 613 |
| p-cox1-c | 6,023 | 6,115 | 93 | 399 |
| H | 6,515 | 6,569 | 55 | 70 |
| K | 6,640 | 6,694 | 55 | 4 |
| Y | 6,699 | 6,752 | 54 | 15 |
| Q | 6,768 | 6,821 | 54 | -10 |
| S2 | 6,812 | 6,874 | 63 | -2 |
| N | 6,873 | 6,928 | 56 | 33 |
| M | 6,962 | 7,017 | 56 | 6 |
| G | 7,024 | 7,077 | 54 | 1 |
| rrnS | 7,079 | 7,752 | 674 | 0 |
| W | 7,753 | 7,808 | 56 | 50 |
| atp6 | 7,859 | 8,446 | 588 | 22 |
| A | 8,469 | 8,524 | 56 | 6 |
| L2 | 8,531 | 8,586 | 56 | 0 |
| E | 8,587 | 8,641 | 55 | 20 |
| S1 | 8,662 | 8,718 | 57 | -1 |
| C | 8,718 | 8,772 | 55 | 33 |
| rrnL | 8,806 | 9,622 | 817 | 21 |
| F | 9,644 | 9,698 | 55 | 87 |
| D | 9,786 | 9,841 | 56 | 60 |
| V | 9,902 | 9,955 | 54 | 1,686 |
| P | 11,642 | 11,696 | 55 | 59 |
| p-nad5 | 11,756 | 12,121 | 366 | 245 |
| I | 12,367 | 12,422 | 56 | 224 |
| L1 | 12,647 | 12,698 | 52 | 106 |
| nad1 | 12,805 | 13,647 | 843 | 4,595 |
