# Supplementary material for: The mitochondrial genome of Globodera ellingtonae is composed of two circles with segregated gene content and differential copy numbers
Source: BMC Genomics. 2016 Sep 5;17(1):706. doi: 10.1186/s12864-016-3047-x (PMC5011991; doi:10.1186/s12864-016-3047-x)
Supplement: Additional file 4: — Primer sequences. (DOCX 18 kb) [file 12864_2016_3047_MOESM4_ESM.docx]

Primers used for cloning, quantitative PCR, and making Southern probes.

| Cloning primers | primer sequence (5'->3') | position on circle | |
| --- | --- | --- | --- |
| mtDNA-I for1 | GGGGTGGATGAAGTTAAGGT | 6735 | 6754 |
| mtDNA-I rev1 | TGAAGGACAACATCCAACCT | 16103 | 16122 |
| mtDNA-I for2 | TTGTTGGGATAGACTTGGAT | 15900 | 15919 |
| mtDNA-I rev2 | GAGAAGGAAATACCTGAAACACC | 7064 | 7086 |
|  |  |  |  |
| mtDNA-II for1 | CGTTCCAGAAGATTCGGCTA | 7183 | 7202 |
| mtDNA-II rev1 | CTTGTTGTCTCTATCGTTCCT | 1681 | 1701 |
| mtDNA-II for2 | GAGTTCGGTTTCGCAGGT | 14138 | 14155 |
| mtDNA-II rev2 | AAAAGACTCAATATCCCCAGAT | 6733 | 6754 |
| mtDNA-II for3 | ATTCTTGGTTGTGGGTCGAG | 6537 | 6556 |
| mtDNA-II rev3 | TCCGATTTCATACCCCCTTA | 7608 | 7627 |
|  |  |  |  |
| Southern probe primers |  |  |  |
| mtDNA-I for | GAGTTCTGGGGATGTTTTATCC | 7029 | 7050 |
| mtDNA-I rev | CCACAAATCTCCGAACACTG | 7431 | 7450 |
| mtDNAII for | GTGTCTAAATACTCTTTGTTGG | 13158 | 13177 |
| mtDNAII rev | CGGAGGACACCTGACTAAA | 13761 | 13779 |
| Shared for^a^ | GGGAGGCAACTTTACAGGATTC | 4754 | 4775 |
| Shared rev | GTAGAAGAAGCCTCACGTGTACC | 5606 | 5628 |
|  |  |  |  |
| qPCR primers |  |  |  |
| mtDNA-I for | GGGTTTCGGTTCTTCTGGA | 9050 | 9068 |
| mtDNA-I rev | CCCAAAACCCAAGATCTAAAGG | 9199 | 9220 |
| mtDNA-II for | TTGGGTTCGGTCCGTCTTTT | 13174 | 13193 |
| mtDNA-II rev | CAAAAGGCCTCCGATTAACCTC | 13339 | 13360 |

^a^Positions for shared primers are relative to mtDNA-I
